# Striatal dopaminergic alterations in Tourette’s syndrome: a meta-analysis based on 16 PET and SPECT neuroimaging studies

**DOI:** 10.1038/s41398-018-0202-y

**Published:** 2018-08-02

**Authors:** Marius Hienert, Gregor Gryglewski, Mara Stamenkovic, Siegfried Kasper, Rupert Lanzenberger

**Affiliations:** Department of Psychiatry and Psychotherapy, Medical University of Vienna, Vienna, Austria

## Abstract

Despite intense research, the underlying mechanisms and the etiology of Tourette’s syndrome (TS) remain unknown. Data from molecular imaging studies targeting the dopamine system in Tourette patients are inconclusive. For a better understanding of the striatal dopamine function in adult dopamine-antagonist-free patients we performed a systematic review in August 2017 identifying 49 PET and SPECT studies on the topic of TS. A total of 8 studies appraised the dopamine transporter (DAT) with 111 Tourette patients and 93 healthy controls, and could be included in a meta-analytic approach. We found a significantly increased striatal DAT binding in Tourette patients (Hedges' *g* = 0.49; 95% CI: (0.01–0.98)), although this effect did not remain significant after correcting for age differences between cohorts. A second meta-analysis was performed for the striatal dopamine receptor including 8 studies with a total of 72 Tourette patients and 71 controls. This analysis revealed a nonsignificant trend toward lower dopamine 2/3 receptor binding in striatum of Tourette patients. Other analyses regarding study population characteristics in both the DAT and receptor meta-analysis did not show any meaningful results. Our results indicate that dopaminergic alterations in TS are likely and thereby this data would be in line with the current pathophysiological hypotheses of a dysfunction in the dopamine system, e.g., the hypothesis of tonic-phasic dysfunction. However, these analyses suffer from low effect sizes probably due to the heterogeneity of TS and highlight the need for further large-scaled neuroimaging studies.

## Introduction

Tourette’s syndrome (TS) is a heritable neuropsychiatric movement disorder, which is clinically characterized by the simultaneous presence of at least one vocal tic and multiple motor tics for at least 12 months^1^. According to the Diagnostic and Statistical Manual of Mental Disorders Fifth Edition’s [DSM-V] the tic onset of TS must be prior to the age of 18^2^. Most Tourette patients have associated neuropsychiatric comorbidities such as obsessive–compulsive disorder (OCD) and attention deficit hyperactivity disorder (ADHD). Those affected also frequently suffer from mood disorders or rage attacks^3^.

Today there are several treatment strategies taking the neurobiological pathophysiology of TS into account^4^. Since the 1970s several classes of psychotropic drugs, especially dopamine-antagonists, have proven their efficacy in reducing frequency and severity of vocal and motor tics. Nonetheless, response rates to pharmacological standard therapy remain unsatisfactory^5^. There are only a limited number of randomized controlled trials for the pharmacological treatment of TS. This lack of evidence for safety and efficacy of pharmacotherapy leads to the absence of firm recommendations. Furthermore, the drugs commonly used are often associated with severe side effects, leading to discontinuation of medication^4,6^. Although there are promising new pharmacological treatment strategies, such as pregabalin^7^ or aripiprazole^8^, there is no causal therapy for TS. Modern pharmacological treatment normally only results in a 50% reduction in tic symptoms^5^. Although the side effect profile of modern therapeutics like aripiprazole seems to be more favorable^8^, the limited knowledge of TS pathophysiology disables the development of a curative therapeutic approach.

Different neuroimaging techniques, electrophysiological studies, animal models, and postmortem studies support the hypothesis of a dysfunction in cortico-striato-thalamo-cortical networks as a neurobiological substrate of tics^9,10^. Many neuroimaging studies using structural and functional magnetic resonance imaging (MRI), positron emission tomography (PET), single-photon emission computed tomography (SPECT), and combined approaches have given us insight into the pathophysiology of TS. Nonetheless, there are still inconsistencies across those studies. Therefore, a comprehensive understanding of the specific neurobiological mechanisms underlying the origin of tics remains incomplete^11^.

The number of new publications on TS is steadily increasing^12^. Especially in the last few years many fMRI studies, estimating brain activity by blood oxygen level dependent signal, were carried out. Two recent meta-analyses attempted to summarize this body of evidence. The first study revealed inhibition control deficits via neuropsychological tasks in TS patients by analyzing the data of 1717 TS patients^13^. The other study focused on the functional circuitry of TS by evaluating task-based neuroimaging studies. They found differences in motor preparation and prefrontal cortices in TS patients by investigating 651 participants^14^.

Early neuroimaging research on TS focused on the basal ganglia and the role of dopamine^15^. These studies were driven by the fact that dopamine receptor blocking agents and dopamine-depleting drugs reduce tics in TS patients^1^. However, postmortem^16–18^ and imaging studies focusing on the pathophysiological hypothesis of striatal dopaminergic hyperinnervation showed inconclusive results^19^. At this point, a meta-analytic approach is needed to synthesize the existing dopamine radiotracer neuroimaging literature to gain a better picture.

Meta-analyses of molecular imaging studies have provided important information for the etiology of other mental disorders. Gryglewski et al.^20^ revealed a widespread reduction of serotonin transporter binding in patients with major depressive disorder (MDD) by investigating 18 PET and SPECT studies. Similar meta-analyses of molecular imaging markers have shed light on the role of dopamine in schizophrenia. Two analyses with large sample sizes showed no alterations in the availability of dopamine transporter (DAT) in schizophrenia^21,22^. Yet a small increase in dopamine receptor availability^22^ and striatal dopamine synthesis capacity could be detected in schizophrenic patients when compared to healthy^23^. These meta-analytic studies have provided clarity about the role of neurotransmitters in MDD and schizophrenia, as one of the main reasons for false-positive findings are small sample sizes and publication bias^24^. Here, we are using similar methods to clarify the role of striatal dopamine in TS.

There are several ligand studies utilizing SPECT or PET to examine the function of the striatal dopamine system, yet a number of these studies failed to show differences between TS patients and controls. This might reflect the poor spatial resolution of the current scanning techniques^25^. PET offers greater sensitivity as well as specificity than SPECT, which tends to have low-specific binding to the target structure^26^. But neuroimaging studies tend to be confounded by other factors such as age, medical treatment, subject matching, and more. This meta-analysis aims to clarify the role of dopamine in TS.

## Materials and methods

### Data collection

A systematic literature search was conducted on PubMed (www.ncbi.nlm.nih.gov/pubmed) in August 2017 using the following search terms: “Tourette” OR “Tourette’s” AND “neuroimaging” OR “PET” OR “positron emission tomography” OR “single-photon emission computed tomography” OR “SPECT”.

### Study selection

Studies were only included in this meta-analysis if they met the following inclusion criteria:

- Studies report means and standard deviation values of PET or SPECT outcome measures reflecting ligands to the cerebral dopamine system.
- Studies published in English in a peer-reviewed journal.
- Studies present original data.
- Studies include a group of human patients suffering from TS and a group of healthy controls.
- Studies include adolescents and adults.

Studies were excluded from the meta-analysis if the:

- Studies included subjects with psychiatric comorbidities (other than comorbid ADHD or OCD) or severe somatic diseases.
- Studies included TS patients undergoing current therapy with dopamine-antagonists and the absence of an at least 3 month long washout period.
- Studies included children.

We contacted the corresponding authors of all eligible studies in this meta-analysis to obtain further information about missing data or their results per e-mail. Two authors responded and were able to provide data needed for a sufficient analysis.

Beside the PET or SPECT outcome for each brain region, we included demographic variables (age and sex), type of outcome measure and tracer, psychotropic drug history (drug-naïve patients and drug-free interval) as well as TS severity in our analyses. Due to the high interrater and interstudy variability in the different Tourette severity scores this approach must be viewed as explorative. Most studies used the Yale Global Tic Severity Scale (YGTSS)^27^ as an instrument for the assessment of tic severity. Only three studies used scores other than the YGTSS, namely the Shapiro Tourette Syndrome Severity Scale^28^ and the Modified Rush Videotape Rating Scale^29^. Due to the small number of other instruments used we treated them as equivalent to the YGTSS in our analysis.

During this systematic search a total of 49 PET and SPECT studies in the field of TS were found. Of those, 19 were excluded because they examined regional brain activity (9 SPECT and 10 PET). Seven studies were excluded because they focused on neurotransmitters other than dopamine (Cannabis, GABA, and VAChT). Another 9 studies could not be included because they included children, used special targets, e.g., VMAT, or results could not be sufficiently used in a meta-analytic approach. In the remaining 14 published studies two types of dopaminergic targets in the striatum were investigated. The first neurotransmitter target of interest was the DAT. The other was the dopamine receptor (D_2/3_).

### Statistical analysis

The statistical analyses were performed as described previously in Gryglewski et al.^20^. The software package R 3.4.1., and for specific meta-analytic computations the metafor package version 2.0-0., were used for analysis^30^.

#### Individual study effect estimates

Principally PET and SPECT studies report a number of different outcome measures. This ranges from the simple uptake ratio in a region of interest compared to a nonbinding reference region to the binding potential (*BP*_*ND*_), a dimensionless quantity which is proportional to the binding parameters *B*_*max*_*/K*_*d*_, when dynamic emission sequences are obtained. Other studies report the tracer equilibrium distribution volume (*V*_*t*_; ml/g), if the arterial input function is available. To use all these different outcome measures in a meta-analytic way, standardized effect size estimates were computed. Therefore, standardized mean difference (Hedges' *g*) for each study and brain region were calculated^31,32^. Hedges' *g* is the difference between the two means which is divided by their pooled standard deviation^33^.

#### Summary effect estimates

We conducted meta-analyses for DAT in the striatum and its subregions as well as cerebral hemispheres, if available. Furthermore, we conducted meta-analyses for D_2/3_ in the striatum and its subregions. We used random-effects models. Restricted maximum likelihood estimation was used to compute resulting findings. In addition, Higgins' *I*^2^, an intuitive measure of study estimate variation due to study heterogeneity^34^, was calculated. To check for publication bias funnel plots were applied to show the precision of the studies against their effect estimates.

#### Influence of study population characteristics

Sensitivity of estimates to age differences, prior medication, sex ratio and tic severity was assessed by mixed-effects models. Due to a number of TS patients with comorbid OCD or ADHD (according to information reported in the analyzed papers) this parameter was not analyzed in a mixed-effects model.

## Results

Fourteen original PET or SPECT studies reflecting ligands to the striatal dopamine system in adult TS patients and matched healthy controls were included in this meta-analysis. Within these studies, six were targeting the DAT while the other six studies investigated the dopamine receptor. Two studies had examined both the dopamine receptor and the transporter in a TS patient group as well as a group of healthy subjects.

Two separate meta-analyses for eight DAT studies and eight dopamine receptor studies were performed. For the analysis of the DAT data from 6 SPECT and 2 PET studies comprising a total of 111 TS patients and 93 healthy controls were included. See Table 1 for a detailed illustration of the selected DAT studies. For the analysis of the dopamine receptor, data from 2 SPECT and 6 PET studies comprising a total of 72 TS patients and 71 healthy controls were included. See Table 2 for a detailed illustration of the selected D2/3 studies. In the following, the results of the two different binding sites are shown. If data for subregions and lateralization was available the results of this subanalysis are shown as well.

**Table 1:** The key data of all studies targeting the dopamine transporter

**Year**	**First author**	**Tracer (target)**	**Method**	**TS patients**	**Healthy controls**	**Scores**	**Drug free**
2000	Müller-Vahl	ß-CIT (DAT)	SPECT	6	9	STSS 3,3 (6)	5 month
2001	Stamenkovic	ß-CIT (DAT)	SPECT	15	10	YGTTS 67,65 (100)	Drug-naïve
2004	Serra-Mestres	FP-CIT (DAT)	SPECT	10	10	YGTTSS 47,6 (100)	Drug-naïve
2007	Yeh	TRODAT (DAT)	SPECT	8	8	YGTTSS 25 (100)	Drug-naïve
2008	Hwang	TRODAT (DAT)	SPECT	10	15	MRVRS 11,7 (20)	3 month
2008	Wong	C11 WIN (DAT)	PET	11	5	YGTSS 50,27 (100)	6 month
2009	Albin	MP (DAT)	PET	33	28	YGTSS 37 (100)	3 month
2010	Liu	TRODAT (DAT)	SPECT	18	8	YGTTSS 39,17 (100)	Drug naive
**SUM**				**111**	**93**		

*Method*: positron emission tomography (PET), single-photon emission computed tomography (SPECT). *Scores*: Yale Global Tic Severity Scale (YGTSS); Shapiro Tourette Syndrome Severity Scale (Shapiro STSS); Modified Rush Videotape Rating Scale (MRVRS). *Drug free*: drug-free interval before scan

**Table 2:** The key data of all studies targeting the dopamine receptor

**Year**	**First author**	**Tracer (Target)**	**Method**	**TS patients**	**Healthy controls**	**Scores**	**Drug free**
1994	Turjanski	RACLOPRIDE (D_2/3_)	PET	5	9	no	3 month
2000	Müller-Vahl	IBZM (D_2_)	SPECT	10	7	STSS 3,4 (6)	12 month
2002	Singer	RACLOPRIDE (D_2/3_)	PET	7	5	YGTSS 38 (100)	6 month
2008	Hwang	I-IBZM (D_2_)	SPECT	11	15	MRVRS 11,7 (20)	3 month
2008	Wong	RACLOPRIDE (D_2/3_)	PET	11	7	YGTSS 50,27 (100)	6 month
2013	Denys	RACLOPRIDE (D_2/3_)	PET	12	12	YGTTS 17 (50)	6 month
2015	Black	RACLOPRIDE (D_2/3_)	PET	5	5	YGTSS 27,8 (100)	Drug naive
2015	Abi-Jaoude	RACLOPRIDE (D_2/3_)	PET	11	11	YGTTS 19,9 (50)	3 month
**SUM**				**72**	**71**		

*Method*: positron emission tomography (PET), single-photon emission computed tomography (SPECT). *Scores*: Yale Global Tic Severity Scale (YGTSS); Shapiro Tourette Syndrome Severity Scale (Shapiro STSS); Modified Rush Videotape Rating Scale (MRVRS). *Drug free*: Drug free interval before scan

### DAT

#### Striatum

**Fig. 1:**
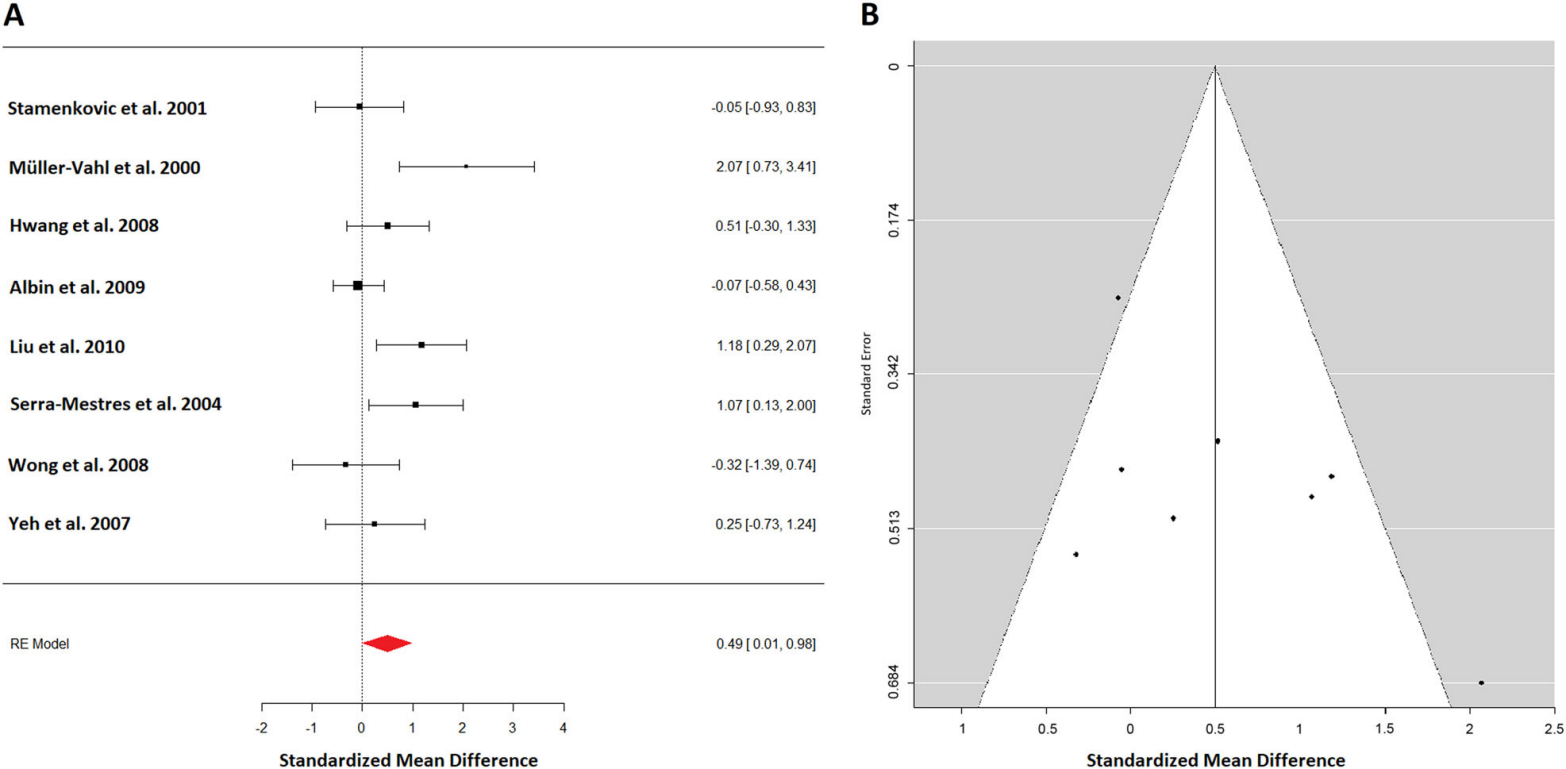
**a** A forest plot of all available dopamine transporter (DAT) studies in TS patients summarizing to an effect size of 0.49 standard deviation, indicating a scarce significant effect of higher dopamine transporter availability in TS patients. **b** A funnel plot showing the precision of all dopamine transporter studies against their effect estimates. It appears symmetrical except for two outliers one at the right bottom and one at the left top

For the analysis of the striatum all available 8 studies with 111 patients and 93 controls could be included (Fig. 1a). Effect estimates showed moderate heterogeneity (*I*^2^ = 58.89%). The summary effect estimates indicated a scarce significant effect of higher DAT availability in TS patients (0.49; 95% CI: (0.01–0.98)). However, when a mixed-effects model was performed correcting for age differences between groups (control groups tended to be older), the effect of age difference became significant and the effect of group differences was not significant anymore. It became half the size (0.27; 95% CI: (−0.16 to 0.7)). The funnel plot for striatal DAT binding appears symmetrical, except for two outliers at the bottom right and top left (Fig. 1b). Leave-one-out analysis revealed no meaningful influence. The remaining analysis concerning population characteristics for gender, and tic severity found no influence of these variables. The effect on drug-naive patients was higher but not significant. For 78 TS patients and 75 healthy controls from 6 studies a separate analysis for right and left striatum could be performed. Effect sizes were similar, however, not significant, due to the lower number of subjects left (0.48; 95% CI: (−0.06 to 1.02)) right (0.38; 95% CI: (−0.03 to 1.06)).

#### Caudate

Six studies in 86 patients and 68 controls appraised the caudate. In this heterogeneous study sample (*I*^2^ = 67.77%) a trend toward a higher DAT availability in TS patients (0.39; 95% CI: (−0.24 to 1.03)) was observed. Leave-one-out analysis indicated omitting the study by Wong et al.^35^ would result in a summary effect size of increased DAT at 0.58 with *p* = 0.056. The mixed-effect model analyses were not significant. We performed a separate analysis for right and left caudate of this dataset. A nonsignificant increase of DAT binding in patients’ left (0.44; 95% CI: (−0.19 to 1.06)) and right (0.34; 95% CI: (−0.31 to 0.99)) caudate remained.

#### Putamen

The same 6 studies with 86 patients and 68 controls reported on the putamen. In spite of the heterogeneous study sample (*I*^2^ = 71.07%) a nonsignificant trend toward a higher DAT availability in TS patients (0.39; 95% CI: (−0.28 to 1.06)) could be seen. Similar to the caudate, omitting data from Wong et al.^35^ resulted in significantly increased DAT binding (*p* = 0.04). Again a separate analysis for right and left putamen was carried out. Once more a nonsignificant increase of DAT binding in patients’ left (0.40; 95% CI: (−0.22 to 1.03)) and right (0.37; 95% CI: (−0.35 to 1.10)) putamen was seen.

#### Summary of DAT results

In the striatum, significantly increased DAT binding in 111 TS patients and 93 controls was detected, but did not remain significant upon correcting for age. In the subanalyses (subregions and lateralization) of this set in every analysis, a nonsignificant trend toward a higher DAT binding in TS patients was observed. Additional analyses did not show any meaningful influences.

### D_2/3_

#### Striatum

**Fig. 2:**
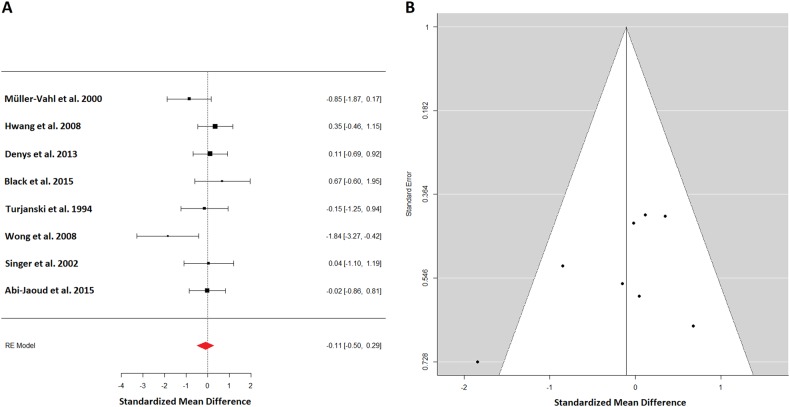
**a** A forest plot for all dopamine receptor (D_2/3_) studies appraising the striatum. The summary effect estimates of −0.11 standard deviation indicated a nonsignificant lower D_2/3_ availability in TS patients. **b** The corresponding funnel plot for dopamine receptor studies of the striatum. It appears symmetrical except for one outlier at the left bottom

Eight studies with 72 patients and 71 controls appraised D_2/3_ in the striatum (Fig. 2a). Effect estimate were mildly heterogeneous (*I*^2^ = 19.00%). The summary effect estimates indicated a nonsignificant alteration of D_2/3_ availability in TS patients (−0.11; 95% CI: (−0.5, 0.29)). The funnel plot for striatal D_2/3_ binding appears symmetrical except for one outlier at the bottom left (Fig. 2b). Leave-one-out analysis revealed no meaningful influence. An analysis for lateralization was not sensible due to data availability from only two studies. The remaining analyses concerning study population characteristics in particular age differences, prior medication and sex ratio found no influence, except for a nearly significant effect of tic-severity correlating with lower D_2/3_ binding in TS patients. This effect was likely due to results from Wong et al.^35^ with severely affected TS patients and significant negative D_2/3_ binding in the patient group.

#### Caudate

Five studies in 40 patients and 38 controls appraised the caudate. In this homogenous study sample (*I*^2^ = 00.00%) summary effect estimates (−0.01; 95% CI: (−0.42 to 0.41)) showed no significant alterations. None of the other performed analyses found any notable effect. No lateralization analysis was possible.

#### Putamen

The same 5 studies with 40 patients and 38 controls reported on the putamen. The study sample was heterogeneous (*I*^2^ = 66.88%) and no significant difference in D_2/3_ availability in TS patients (−0.19; 95% CI: (−0.95 to 0.57)) was observed. None of the other analyses performed showed a meaningful influence of effect estimates in the putamen. Again, no differentiation between hemispheres could be observed.

#### Summary of D_2/3_ results

A nonsignificant trend towards lower D_2/3_ binding in striatal regions of 72 TS patients compared to 71 healthy controls could be observed. Furthermore, a trend toward lower D_2/3_ binding and higher tic scores was detected. Other analyses did not show any meaningful results.

## Discussion

Our results suggest increased DAT binding in TS. Studies in healthy subjects have shown a decline of striatal DAT availability with aging, losing about 8% DAT binding per decade^36,37^. This highlights the importance of appropriate matching of subjects, especially if the outcome of interest is known to be dependent on a certain demographic parameter. In our dataset big age differences in groups could be found in the studies of Wong et al.^35^ and Müller-Vahl et al.^38^. This might be the reason why the leave-one-out analysis performed for caudate and putamen indicated omitting the study of Wong et al.^35^. The studies included in this meta-analyses scanned population of varying ages. TS is a neurodevelopmental disorder starting in childhood^39^. It is suggested that dopaminergic innervation of the striatum peaks in preadolescence and subsequently decreases during adulthood^40^. Additionally, TS is a very heterogeneous disorder with different subtypes. It is possible DAT alterations are present at one age and disappear during development and aging, or DAT alterations refer to one exclusive group of TS patients^19^. There are other limitations, which could explain the conflicting results in previous DAT literature on TS^41^. In some of our analyzed studies gender matching as well as equal group size were insufficient. An international study screened 3500 TS patients and reported massive variations in symptom severity and frequency over time giving weight to the natural waxing and waning of tics^42^. Most TS neuroimaging studies suffer from small subject numbers, unbalanced gender ratios, improper TS staging, interferences of former drug treatment, the aforementioned heterogeneity of clinical severity in TS patients^39^, as well as differences in scan techniques and data analysis methodology^43^. These causes may also be a considerable reason why additional analyses, e.g., for tic severity, did not return any meaningful results.

For D_2/3_ receptor binding a nonsignificant trend toward lower availability in TS patients was observed. Again the aforesaid reasons regarding the discrepant findings in TS neuroimaging literature have to be mentioned. There are three mechanisms that could explain the negative trend: more endogenous dopamine which competes with the tracer, fewer affinity and/or density to the dopamine receptor or both mechanisms^43^. The possible pathophysiological hypothesis will be discussed later. Furthermore, a nearly significant effect of tic-severity and lower D_2/3_ receptor binding in TS patients was observed. This would suggest that lower D_2/3_ receptor levels are indeed a pathological characteristic of TS^43^. But this correlation was very likely due to the data of Wong et al.^35^. This study included severely affected TS patients and had significant results indicating lower dopamine receptor binding in TS.

### Tourette’s pathophysiological hypotheses

The hypothesis of dopamine dysfunction in TS patients was first driven by the treatment success of dopamine blocking agents^5^. It was supported by neuroimaging findings and postmortem brain studies. Over time four different hypotheses of dysfunction in the dopamine system have evolved^44^: the hyper-innervation, the supersensitive receptor, the presynaptic abnormality, and the tonic-phasic dysfunction dopamine hypothesis of TS.

### DAT

Our findings of higher DAT binding in adult TS patients can test the hypothesis of hyperinnervation and the presynaptic abnormality hypothesis. The DAT is located in presynaptic dopamine neurons and responsible for the dopamine reuptake from the synaptic cleft, thereby controlling extracellular dopamine levels^45^. The hyperinnervation hypothesis states that TS and tics in particular are caused by an excessive or more precisely an over innervation of dopamine terminals especially in the striatum^44^. The presynaptic dopamine abnormality hypothesis in TS states a more general alteration of presynaptic dopamine neurons^46^. Both ideas were supported by postmortem brain tissue studies. Two studies showed an increase of DAT in the brain of TS patients compared to matched controls^16,17^. So far, neuroimaging data on DAT in TS has been inconsistent^46^. The results of our analysis reduce the often mentioned problem of small subject number, heterogenic population samples (age, tic severity, and medication history) and differences in imaging technique by pooling the available data. Another limitation for consequent conclusions from these findings is the chicken-and-egg problem^44^. Bigger DAT density in TS patients may reflect a causal pathophysiological mechanism in tic-generation, potentially by striatal dopaminergic hyper-innervation, or it reflects an adaptive change caused by the disease or pharmacological treatment. At this point, both mechanisms seem to be possible according to the available literature.

### D_2/3_

Our findings of a trend toward lower D_2/3_ receptor binding in adult TS patients can test for the hypothesis of supersensitive dopamine receptors in TS. This hypothesis states a postsynaptic abnormality of dopamine receptors due to changes of affinity and/or density of those receptors^44^. There are two different G protein-coupled receptor types which mediate dopamine neurotransmission^47^: the excitatory D_1_ type and the inhibitory D_2_ type^44^. D_2_-like receptors include the dopamine receptor 2, 3, as well as 4, and D_1_-like receptors include 1 and 5^48,49^. Most dopamine receptor-expressing neurons in the human brain are GABAergic medium spiny neurons (MSNs), which present 95% of striatal neurons^50^. In TS patients the cortico-basal ganglio-thalamo-cortical (CBGTC) network is altered^51,52^. These alterations include both structural and functional changes of the CBGTC network involving several reentry pathways^53^. In the CBGTC circuit the striatum acts as an input center for the basal ganglia from the cortex. While the so called hyperdirect pathway bypasses the striatum, striatal efferents are classified as direct and indirect pathways. The indirect pathway expresses D_2_ receptors, which project to the globus pallidus and the substantia nigra, polysynaptically^54^. Our above-mentioned results with a trend towards a lower D_2/3_ receptor affinity and/or density are not in line with the classic supersensitive dopamine receptor hypothesis, where a higher binding of receptors would have been expected. The inconsistencies of previous neuroimaging studies could be overcome with this meta-analytic approach. Several PET and SPECT studies showed no D_2/3_ receptor differences between TS patients and controls. Accordingly, earlier postmortem findings did not show differences between dopamine receptors in TS brains and those of healthy controls. These findings in the brains of TS patients were limited by a very small sample size^16–18^. Whereas two PET studies, which assessed extrastriatal D_2/3_ receptors in a set of six^55^ and eight TS patients^56^ found a significant reduction of dopamine receptor density compared to matched healthy controls. Lower D_2/3_ receptor availability or affinity (hyposensitive dopamine receptors) may be one of the causal mechanisms of TS. But again, the chicken-and egg dilemma confines this statement^44^. Also an adaptive mechanism, potentially due to drug treatment, may be the reason for decreased receptor availability or once again both reasons may be accountable for these postsynaptic changes.

### Tonic-phasic model of dopamine

Another popular pathophysiological hypothesis of TS states a dysfunction of the tonic-phasic dopamine release^57^. The recent model of dopamine function states the regulation of dopamine activity in subcortical regions operates via two mechanisms^58^. First, the tonic dopamine release, where dopamine is released into the extracellular space in low concentrations via different mechanisms continuously and second, the spike-dependent phasic dopamine release. In the phasic state dopamine is released in high concentration into the synaptic cleft and rapidly inactivated^59^. The classic tonic-phasic TS theory suggests that an overactive DAT would cause reduced tonic dopamine transmission. This in turn reduces autoreceptor availability and increases presynaptic dopamine levels due to higher dopamine reuptake. This would result in an increased phasic dopamine release^60^. Our findings of higher DAT and lower D_2/3_ availability fit well with this theory. However, this classic hypothesis of tonic-phasic dysfunction has an obvious problem. Psychostimulants which inhibit the DAT do not reduce vocal or motor tics. Therefore, Maia and Conceição developed a new hypothesis of tonic-phasic dysfunction in TS, which involves an increase in tonic and phasic dopamine^57^. This theory is grounded on the computational understanding of action selection and learning in striatal dopamine function. So the CBGTC network and striatal dopamine are involved in tic execution and especially phasic mediated tic learning^61^. In the striatum dopamine inhibits D2 MSNs of the indirect pathway and stimulates the activity of D1 MSNs of the direct pathway^62^. Maia and Conceição postulate a striatal dopaminergic hyperinnervation in patients with TS^57,61,63^. Our findings are in line with this new approach and can serve as a neuroimaging underpinning of this hypothesis.

## Conclusion

Neuroimaging studies on the topic of TS have reported inconclusive results. To gain a better picture we performed the to our knowledge first meta-analysis on molecular imaging of the dopamine system in TS. A systematic literature search in August 2017 found a total of 49 PET and SPECT studies in the field of TS. For investigating the DAT activity 8 studies with 111 patients and 93 healthy controls could be included in our meta-analysis. Significantly increased striatal DAT binding in TS patients was detected, although this effect did not remain significant after correcting for age. For the dopamine receptor activity analysis of 8 studies with a total of 72 TS patients and 71 controls revealed a nonsignificant trend towards lower striatal D2/3 receptor binding in TS patients. Our data suggests striatal dopaminergic alterations in adult TS patients, which emphasizes the current hypotheses of TS pathophysiology. However, the here observed effects were nonsignificant or did not remain significant after correcting for covariates. This might highlight the heterogeneity and age dependency of TS. Nonetheless, our data clearly demonstrate the need for large-scaled, systematic, longitudinal neuroimaging studies with well-matched control groups to understand the pathophysiology of TS and thereby make the development of a curative therapy possible.
